# Comparison of radiation dose and image quality between flat panel computed tomography and multidetector computed tomography in a hybrid CT‐angiography suite

**DOI:** 10.1002/acm2.12808

**Published:** 2020-01-10

**Authors:** Aaron K. Jones, Bruno C. Odisio

**Affiliations:** ^1^ Department of Imaging Physics The University of Texas MD Anderson Cancer Center Houston TX USA; ^2^ Department of Interventional Radiology The University of Texas MD Anderson Cancer Center Houston TX USA

**Keywords:** cone beam CT, flat panel CT, multidetector CT

## Abstract

The purpose of this study was to compare, using the same radiation dose and image quality metrics, flat panel computed tomography (FPCT) to multidetector CT (MDCT) in interventional radiology. A single robotic angiography system with FPCT was compared to a single MDCT system, both installed in a hybrid CT‐angiography laboratory and both operating under automatic exposure control. Radiation dose was measured on the central axis (D_c_) of a CT dosimetry phantom 30 cm in diameter and 60 cm in length using default protocols for FPCT and MDCT with the imaged length in MDCT matched to the field of view of FPCT. The noise power spectrum (NPS), modulation transfer function (MTF), and z‐axis resolution were measured using the same phantom. Iodine contrast to noise ratio (CNR) was also measured. Radiation dose (D_c_) was 41%–69% lower in MDCT compared to FPCT when default protocols and automatic exposure control were used. While spatial resolution could generally be matched with appropriate choice of kernel in MDCT, MTF dropped more quickly at higher spatial frequency for MDCT than FPCT. Image noise was 49%–120% higher for MDCT compared to FPCT for comparable in‐plane spatial resolution. Z‐axis resolution was slightly better for MDCT than FPCT, while iodine CNR depended on protocol selection. Radiation dose was much lower for MDCT compared to FPCT, but image noise was much higher. Matching image noise in MDCT to FPCT would result in similar radiation doses. Iodine contrast depended on dose modulation settings for MDCT.

## 1 Introduction

Volumetric X‐ray imaging is commonly used during fluoroscopically guided interventional procedures (FGI), for tasks such as mapping of vasculature, identification of occult lesions, identification of tumor‐feeding vessels, and verification of therapeutic endpoint.^1^, ^2^, ^3^, ^4^, ^5^, ^6^ Historically, intraprocedural imaging during FGI was performed using flat panel computed tomography (FPCT), also known as cone beam computed tomography (CBCT)^1^. Recently, hybrid computed tomography (CT)‐angiography systems have become available as an alternative to standalone angiographic systems. With increasing installations of hybrid CT‐angiography systems in hospitals over the last several years, questions regarding the comparative performance of conventional multidetector CT (MDCT) and flat panel computed tomography (FPCT) have become more common. Technical, clinical, and financial differences exist between MDCT and FPCT and these differences may affect the utility of the technologies for intraprocedural imaging during fluoroscopically guided interventions.

Previous investigators have used different methods in attempts to answer these questions. Steuwe et al. compared FPCT to MDCT for endovascular aneurysm repair (EVAR) and found effective doses (*E*) of 4.9 mSv for FPCT compared to 2.6 mSv for MDCT for single phase imaging with matched imaged length and both scans under automatic exposure control (AEC).^7^ Bai et al. measured *E* of 7.04 mSv for the FPCT compared to 8.42 mSv for MDCT using fixed technique at 120 kV, and reported lower contrast‐to‐noise ratio (CNR) and higher noise for FPCT compared to MDCT.^8^ Kwok et al. compared FPCT to a fixed technique MDCT protocol and measured *E* of 15 mSv for FPCT compared to 9.8 mSv for MDCT.^9^ In light of these discordant results, further study is needed to characterize the comparative performance of FPCT and MDCT.

The goal of the present work was to conduct, to the extent possible, an apples‐to‐apples comparison of the technical performance of PCT and MDCT in terms of radiation dose and image quality.

## 2 Methods

Radiation dose and image quality were compared between FPCT on an Artis zeego angiography system (Siemens Healthineers, Malvern, PA) and conventional MDCT on a Definition Edge Sliding Gantry (SG) CT (Siemens Healthineers, Malvern, PA) using the same phantoms and methods. Both systems were installed in the same hybrid CT‐angiography laboratory. Institutional Review Board approval was not required for this phantom study.

### 2.1 Radiation dose

Radiation dose was measured using the prototype International Commission on Radiation Units and Measurement (ICRU) phantom, a high density polyethylene (HDPE) cylinder 60 cm long and 30 cm in diameter. A 0.6‐cc Farmer‐type ionization chamber (Radcal Corporation, Monrovia, CA) was used to measure the central axis dose (D_c_) in the phantom according to the methods developed and published by American Association of Physicists in Medicine Task Group 111.^10^ The default 6sDCT Body and 5sDCT CARE Body FPCT organ programs on the angiography system were used to image the phantom, which was positioned at isocenter. The acquisition parameters for these programs are listed in Table 1, and the fluoroscope acquired projection images using AEC, as is standard during clinical operation. The default Abdomen Routine CT protocol was used to measure radiation dose (D_c_) for MDCT in the same fashion. The prototype ICRU phantom was positioned at isocenter and a topogram of the phantom was acquired. The Abdomen Routine protocol used both tube current (CareDose 4D) and kV modulation (CarekV, Siemens Healthineers, Malvern, PA). The CarekV algorithm is task‐specific,^11^ and for the current study Slider Position 7 (soft tissue contrast) and Slider Position 9 (midway between soft tissue contrast and vascular) were evaluated. The length of the scan was set to provide the same *imaged* length in MDCT as was acquired using FPCT. The acquisition parameters for the MDCT scan are listed in Table 2. These methods allowed for comparison of radiation dose on an interval scale.

**Table 1:** Acquisition parameters for FPCT.

Organ program	kV plateau	Pulse width (ms)	Min. filtration (mm Cu)	Max. filtration (mm Cu)	Focus	Dose per frame (µGy)	Extended pixel depth	Resolution^a^	Bit depth	3D angle (°)	3D step (°/frame)
5sDCT CARE Body	90	5	0.0	0.0	Large	0.36	On	Low	16	200	0.8
6sDCT Body	90	5	0.0	0.0	Large	0.36	On	Low	16	200	0.5

FPCT, flat panel computed tomography.

a Low resolution means detector pixels were binned 4 × 4 during acquisition.

**Table 2:** Acquisition parameters for MDCT.

Protocol	kV	Rot. time (s)	Pitch	Tube current (mA)	Detector configuration	Adaptive dose shield
Abdomen Routine	CarekV (ref. kV = 120, Slider Position = 7 or 9).	0.5	0.6	CareDose 4D, varied	128 × 0.6 mm	On

MDCT, multidetector CT.

### 2.2 Image quality

Image quality was assessed using the American College of Radiology (ACR) CT accreditation phantom and a multienergy CT quality control (QC) phantom (CT ACR 464 and Multi‐Energy CT Phantom, Sun Nuclear Corporation, Melbourne, FL). The software developed by Friedman et al.^12^ was used to calculate modulation transfer functions (MTF) and noise power spectra (NPS) using images of the ACR CT accreditation phantom. It was not possible to use the outer phantom contour to calculate the MTF as described by Friedman et al., as the field of view (FOV) for FPCT was too small. Instead, the air object within the phantom was used to calculate MTFs for both FPCT and MDCT. NPS and standard deviations were measured in the uniform section of the phantom, NPS using the methods of Friedman et al.^12^ and standard deviations using 15 cm^2^ regions of interest (ROI). These methods allowed for comparison of both high contrast spatial resolution and noise on interval scales.

Two objects in the multienergy CT QC phantom simulating different mixtures of iodine contrast and blood, with iodine concentrations of 2 mg/cc and 4 mg/cc, were used to measure iodine contrast and contrast‐to‐noise ratio (CNR). The phantom was scanned twice for each scenario, and the 2 mg/cc object was moved between the periphery of the phantom and the center of the phantom during alternating scans. These methods allowed for comparison of contrast on an interval scale.

Two 0.28 mm tungsten carbide beads in the ACR CT accreditation phantom, one near isocenter and one near the edge of the phantom, were used to compare z‐axis resolution. Measuring the slice sensitivity profile (SSP) or z‐axis MTF was not practical given that FPCT images could only be reconstructed in contiguous (nonoverlapping) images, therefore, the SSP could not be sufficiently sampled. Instead, an approach was taken that allowed for general comparison of z‐axis resolution between FPCT and MDCT. This was accomplished by counting the number of images along the z‐axis in which the signal from the bead was two standard deviations above the mean of a 25 voxel neighborhood surrounding the bead in the two scans of the phantom acquired previously for MTF and NPS calculation. This method, given the differences in image characteristics between FPCT and MDCT, only allowed for comparison on an ordinal scale.

The reconstruction parameters used during the image quality comparison are listed in Table 3. The angiography system used for FPCT had a recent 3D calibration (within 3 months) and the system was running software version VD11B. The XWP reconstruction station was running software version VD10E. The MDCT was running software version VA48A.

**Table 3:** Reconstruction parameters for image quality characterization.

Technology	Organ program	Matrix size	Voxel size (mm) for MTF	Voxel size (mm) for NPS	Reconstruction algorithm/kernel
FPCT	5sDCT Body CARE	512 × 512	0.148^3^	0.476^3^	HU/Normal
FPCT	6sDCT Body	512 × 512	0.148^3^	0.476^3^	HU/Normal
MDCT	Abdomen Routine	512 × 512	0.148^2^ × 0.6 mm^a^	0.469^2^ × 0.6 mm^a^	B30f, B45f, B70f

FPCT, flat panel computed tomography; MDCT, multidetector CT; MTF, modulation transfer function.

^a^ Pixel size x image thickness

## 3 Results

**Figure 1:**
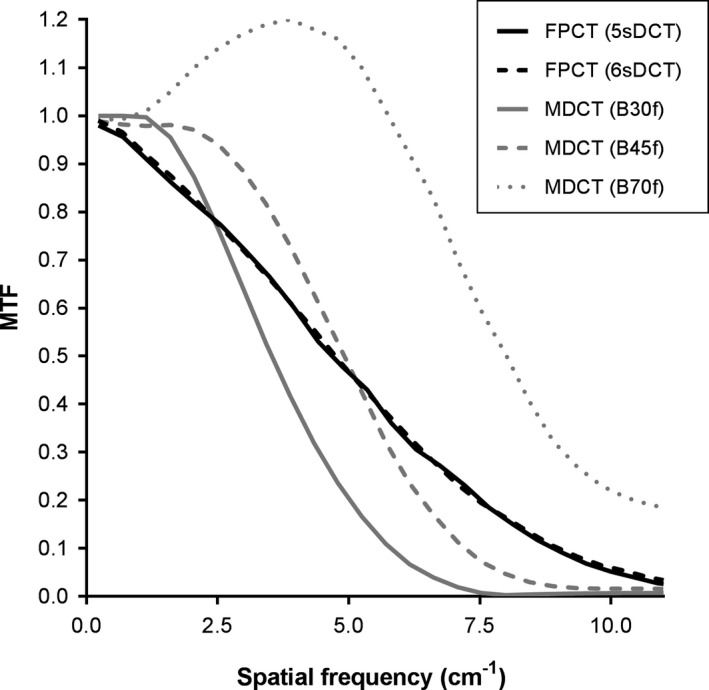
Measured MTF for FPCT and MDCT. MDCT, multidetector CT; MTF, modulation transfer function.

**Figure 2:**
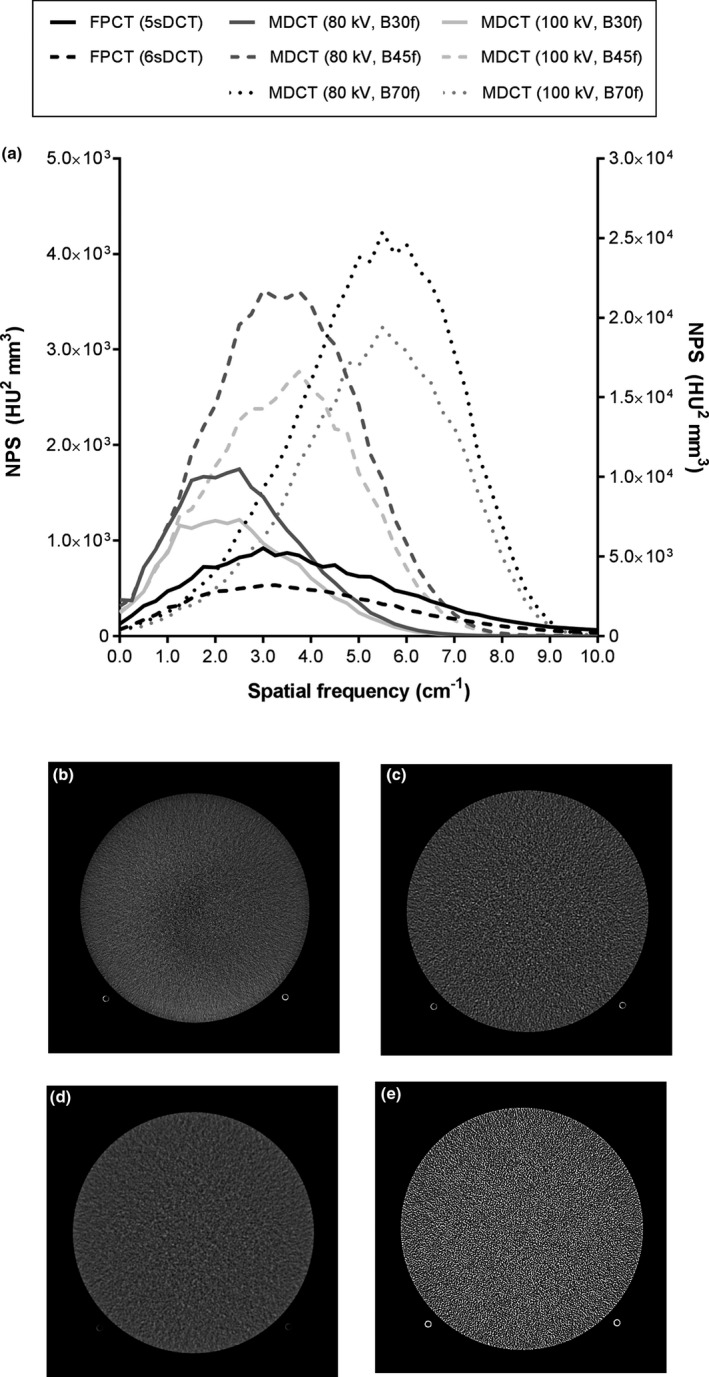
a) Radially averaged NPS; b) image of uniform area of ACR phantom acquired using 6sDCT FPCT protocol; same image acquired using MDCT c) Slider Position 7: 80 kV, B45 kernel; d) Slider Position 7: 80 kV, B30 kernel; e) Slider Position 7: 80 kV, B70 kernel. Note the differences in noise texture among FPCT and MDCT using different reconstruction kernels, and compare to the shapes of the NPS in part a). MDCT, multidetector CT.

Results are summarized in Tables 4 and 5 and Figs 1 and 2. The average mA during FPCT imaging of the ICRU dosimetry phantom was 504 mA and 489 mA for the 6sDCT Body and 5sDCT CARE Body organ programs, respectively. The kV remained at 90 during imaging of the ICRU phantom for both organ programs. The number of projections acquired was 397 and 248 for the 6sDCT Body and 5sDCT CARE Body organ programs, respectively. The mean central axis dose (D_c_), reference air kerma (K_a,r_) and kerma area product (P_KA_) for 6sDCT Body was 32.36 mGy, 128 mGy, and 38.670 Gy‐cm^2^ and for 5sDCT CARE Body was 20.32 mGy, 80.6 mGy, and 24.256 Gy‐cm^2^.

**Table 4:** Measured 0.5 and 0.1 MTF values for FPCT and MDCT.

	5sDCT Body CARE (cm^‐1^)	6sDCT Body (cm^−1^)	Abdomen Routine B30f (cm^−1^)	Abdomen Routine B45f (cm^−1^)	Abdomen Routine B70f (cm^−1^)
0.5 MTF	4.68	4.75	3.54	4.92	8.01
0.1 MTF	8.91	9.00	5.80	7.22	14.9

FPCT, flat panel computed tomography; MDCT, multidetector CT; MTF, modulation transfer function.

**Table 5:** Comparison of iodine contrast and CNR, listed as contrast (HU)/ CNR.

	5sDCT Body CARE	6sDCT Body	Abdomen Routine (Slider Position 7)^a^	Abdomen Routine (Slider Position 9)^a^
4 mg/mL blood/iodine^b^	212.9/7.35	207.7/9.62	210.0/3.37	167.4/2.91
2 mg/mL blood/iodine (central)	113.4/3.01	118.8/3.84	129.2/1.67	105.5/1.56
2 mg/mL blood/iodine (peripheral)	125.8/3.67	123.3/4.93	132.6/1.93	107.8/1.70

MDCT, multidetector CT.

Comparison of radiation dose and image quality between flat panel computed tomography and multidetector computed tomography in a hybrid CT‐angiography suite.

a Slider Position 7 resulted in selection of 80 kV by the MDCT, Slider Position 9 resulted in selection of 100 kV by the MDCT.

b Designed to mimic blood/iodine mixture at the specified iodine concentration.

The technical factors selected by the MDCT based on the topogram with CarekV set to Slider Position 7 were 100 kV and effective mAs of 189 and at Slider Position 9 were 80 kV and effective mAs of 340. To ensure adequate signal was collected in the Farmer chamber, the phantom was scanned at 400 mAs (100 kV) or 500 mAs (80 kV) and the resulting measurements were scaled back to the scanner selected mAs values. While this required the system to use the large focus, the difference in output measured between the small focus, which would be selected based on the parameters selected by the system under AEC, and the large focus was negligible when the same technical factors were used. To achieve an imaged length of 16 cm along the z‐axis of the phantom, matching the FPCT z‐axis FOV at isocenter, a scan length of 17.5 cm was necessary at a pitch of 0.6. When imaged using these parameters, D_c_, CTDI_vol_, and DLP were12.02 mGy, 7.48 mGy, and 130.9 mGy‐cm for the Abdomen Routine protocol using Slider Position 7. When using Slider Position 9, D_c_, CTDI_vol_, and DLP were 10.04 mGy, 6.34 mGy, and 110.8 mGy‐cm, respectively.

Similar values of 0.5 MTF could be achieved for MDCT compared to FPCT by appropriate choice of reconstruction kernel in MDCT: 4.68 cm^−1^ and 4.75 cm^−1^ for 5sDCT CARE Body and 6sDCT, respectively, compared to 4.92 cm^−1^ for the B45 kernel in MDCT (Table 4). There were some differences in the shape of the MTF curves, including evidence of less apodization (i.e., higher MTF at higher spatial frequencies) in FPCT compared to MDCT (Table 4 and Fig. 1).

In general, FPCT was characterized by lower noise than MDCT when technical factors were selected automatically by the imaging systems [Fig. 2(a)]. Comparison of the NPS revealed differences between FPCT and MDCT, including less apodization in FPCT leading to a wider noise “bandwidth” and more peaked NPS in FPCT compared to MDCT. These physical differences are manifested as differences in noise texture between FPCT and MDCT [Fig. 2(b)–(d)]. Standard deviation measurements in the uniform section of the ACR phantom corresponded as expected to the measured NPS, with a larger area under the NPS corresponding to a higher standard deviation. When using the B45f kernel for MDCT the standard deviation was 68.1 at 80 kV (Slider Position 7) and 57.7 at 100 kV (Slider Position 9). For the B30f kernel, standard deviations were 38.1 (Slider Position 7) and 32.3 (Slider Position 9), and for the B70f kernel were 221.5 and 190.4, respectively. For FPCT, the standard deviation was 38.8 for the 5sDCT program and 30.9 for the 6sDCT program.

FPCT was characterized, in general, by lower iodine contrast than MDCT at 80 kV (Slider Position 7) and higher iodine contrast than MDCT at 100 kV (Slider Position 9) (Table 5). Owing to lower noise levels, CNR was higher in FPCT than MDCT. Both contrast and CNR were lower in the center of FPCT images than in the periphery.

Z‐axis resolution was highest for MDCT, with 5sDCT Body CARE being second best and 6sDCT Body the lowest. The nominal voxel size in the z‐axis was 0.6 mm for MDCT, 0.50 mm for 5sDCT, and 0.49 mm for 6sDCT. For both FPCT and MDCT, z‐axis resolution was slightly better at the center of the image than at the periphery. Given the limitations discussed in the Methods section, it was impossible to provide absolute numerical values for z‐axis resolution.

## 4 Discussion

While both FPCT and MDCT are volumetric X‐ray imaging modalities, the results of this study highlight fundamental differences in image quality performance and radiation dose between the two modalities. These differences present unique challenges when attempting to compare the two. To the extent possible, an apples‐to‐apples comparison of FPCT and MDCT was conducted using the stated methods. The modes of operation and protocols provided for clinical imaging were used for both FPCT and MDCT, including AEC, and the imaged length was exactly matched for both modalities.

Radiation dose, quantified as the central axis dose (D_c_), was 41%–69% lower for MDCT compared to FPCT when using default volumetric imaging protocols on both scanners under AEC.

In‐plane spatial resolution can be tailored by the selection of reconstruction kernel in MDCT, and a kernel that matched FPCT closely for 0.5 MTF was identified, B45f. Slight differences in the shape of the MTF remained, likely caused by differences in projection filtering and apodization and perhaps further processing of projection data in FPCT. FPCT transferred more contrast at higher spatial frequencies (i.e., the MTF was higher at higher spatial frequencies, Fig. 1), which would tend to improve the detection of small contrast‐filled vessels and small hypervascular lesions. Higher resolution has been reported for FPCT when reduced or no pixel binning is used,^13^ however, reduced pixel binning increases detector readout time, and therefore increases the data acquisition time and likelihood of patient motion. Z‐axis resolution was the highest for MDCT, which is not surprising considering the use of flying focal spot technology by this model of MDCT to increase sampling and therefore resolution along the z direction.

Overall image noise was much lower for FPCT compared to MDCT when both systems were allowed to set technical factors automatically. This difference is multifactorial. The wide‐area X‐ray beam used in FPCT results in a high scatter‐to‐primary ratio (SPR), even with the use of an antiscatter grid. This scatter radiation tends to reduce noise while also reducing contrast.^14^, ^15^ The exposure control method used for MDCT imaging in this study, CarekV, is designed to optimize the CNR, not just maintain a target image noise level. This is evident in the selection of different kV depending on the imaging task, in this study 80 kV for Slider Position 7 (soft tissue contrast) and 100 kV for Slider Position 9 (midway between soft tissue contrast and vascular). Of course, noise can be scaled easily via the selection of baseline settings for tube current modulation in MDCT (quality reference mAs for the model of CT studied in this work). The data from this study indicate that the quality reference mAs would need to be increased by approximately a factor of 2–3 above the default settings to match the overall noise magnitude in FPCT. This would result in a corresponding doubling or tripling of D_c_, increasing the radiation dose from MDCT to a level that is approximately equal to that from FPCT. Differences in contrast and CNR between FPCT and MDCT resulted from differences in kV and scatter‐to‐primary ratio (SPR), and, as expected, contrast and CNR were better at the periphery than the center of FPCT images.

The results of this study align most closely with those of Stuewe et al.,^7^ indicating that, when using clinical modes of acquisition, radiation doses from FPCT are 100–200% higher than those from MDCT. Kwok et al. found that radiation doses during abdominal imaging using fixed techniques were about 50% higher in FPCT compared to MDCT.^9^ The results of this study are in contrast to those of Bai et al., who found that when using fixed techniques radiation doses from MDCT were 20% higher than FPCT.^8^ Measured MTF values in this study (0.5 MTF and 0.1 MTF, Table 4) matched very closely with values reported by Bai et al. for the 8‐second (8sDCT) FPCT program they studied,^8^ however, comparison of MTF values from MDCT was not possible as Bai et al. did not report the kernels used for MDCT image reconstruction.

Among all the details of this technical comparison, it must not be forgotten that volumetric X‐ray images are often used differently during FGI than for diagnostic imaging. Frequently, the volumetric datasets are used to reconstruct thick multiplanar reconstructions (MPR), maximum intensity projections (MIP), or 3D renderings. Such postprocessing techniques may alter the impact of fundamental image quality characteristics such as noise and MTF on observer perception and performance. Furthermore, this study, as it used phantoms, did not evaluate the influence of the patient on image quality in MDCT and FPCT. These influences include the limited FOV in FPCT compared to MDCT, which limits the volume of interest that can be imaged in some patients; a longer rotation time in FPCT compared to MDCT, which increases the likelihood of motion artifacts; reduced projection sampling in FPCT which leads to streak artifacts, for example, from iodine contrast or embolization coils; and the sampling of all projections along the z‐axis in a single rotation in FPCT, which can result in contamination of the entire imaged volume if patient motion occurs during the scan. A recent study has compared the clinical image quality characteristics of FPCT to MDCT for intraprocedural planning for hepatic transarterial chemoembolization.^16^

This study had several limitations. Radiation dose was not measured on the peripheral axis of the dosimetry phantom, as the FOV for FPCT was too small. This method is acceptable for characterizing the average radiation dose at the center of the scan volume, but ignores the nonuniform surface distribution of radiation dose resulting from limited angle scanning in FPCT and the impact of helical pitch in MDCT. The method used to characterize z‐axis resolution did not allow for numerical quantification of resolution. Third, only filtered back projection (FPB) reconstruction was considered. Iterative reconstruction algorithms are not available for clinical implementations of FPCT, and are not commonly found on MDCT equipment used in interventional radiology. However, iterative reconstruction has been shown to offer the opportunity to reduce radiation dose in MDCT by 10–73% compared to FBP depending on the task,^17^, ^18^ and model‐based reconstruction may offer dose reduction from 47–89% compared to FPB, depending on the task.^19^ While iterative reconstruction and model‐based reconstruction have spatial resolution performance that is similar to FBP for high contrast tasks, their performance is inferior to FPB for low‐contrast tasks at reduced doses.^17^, ^19^ Finally, only a single model of FPCT and MDCT, from a single manufacturer, was studied.

## 5 Conclusion

When a single robotic angiographic C‐arm with FPCT capability and a single MDCT system, both from a single manufacturer and installed in a hybrid angiography laboratory,were operated under automatic selection and optimization of technical factors, noise was much higher in MDCT compared to FPCT, while radiation dose was much lower for MDCT compared to FPCT. However, if noise magnitude is matched, the radiation doses from MDCT and FPCT would be expected to be similar. Spatial resolution was similar between MDCT and FPCT when a suitable reconstruction kernel was selected for MDCT, however, FPCT had slightly higher spatial resolution at higher spatial frequencies. Contrast and CNR were similar between the two modalities. Z‐axis resolution was slightly better for MDCT compared to FPCT. In light of these results, it is reasonable to consider that other differences between MDCT and FPCT, such as the larger FOV, faster data acquisition time, and increased projection sampling in MDCT compared to FPCT, may be more important than the differences in fundamental image quality and radiation dose metrics between the modalities. It is not clear to what extent these results can be generalized, as the FPCT and MDCT systems studied were from a single manufacturer and were operated under automatic exposure control.

## Conflicts of Interest

This work was funded in part by a contract with Siemens Medical Systems, Inc. which is a declared Conflict of Interest for A. Kyle Jones. Bruno C. Odisio has a contract with Siemens Medical Systems, Inc. that is not related to the present work.

## Note

1 We refer to this technology as flat panel computed tomography (FPCT), as all modern CT scanners are, in fact, cone beam CT systems, using a wide detector array along the z axis, and therefore a large cone angle, for imaging. The term cone beam CT (CBCT) is no longer specific to FPCT technology implemented on an interventional C‐arm.
